# Prediction of protein subplastid localization and origin with PlastoGram

**DOI:** 10.1038/s41598-023-35296-0

**Published:** 2023-05-24

**Authors:** Katarzyna Sidorczuk, Przemysław Gagat, Jakub Kała, Henrik Nielsen, Filip Pietluch, Paweł Mackiewicz, Michał Burdukiewicz

**Affiliations:** 1 Faculty of Biotechnology, University of Wrocław, 50-383 Wrocław, Poland; 2 Faculty of Mathematics and Information Science, Warsaw University of Technology, 00-662 Warsaw, Poland; 3 Department of Health Technology, Technical University of Denmark, 2800 Kgs. Lyngby, Denmark; 4 Institute of Biotechnology and Biomedicine, Autonomous University of Barcelona, 08193 Cerdanyola del Vallés, Spain; 5 Clinical Research Centre, Medical University of Białystok, 15-089 Białystok, Poland

**Keywords:** Computational platforms and environments, Machine learning, Protein analysis, Software, Computational biology and bioinformatics, Plant sciences

## Abstract

Due to their complex history, plastids possess proteins encoded in the nuclear and plastid genome. Moreover, these proteins localize to various subplastid compartments. Since protein localization is associated with its function, prediction of subplastid localization is one of the most important steps in plastid protein annotation, providing insight into their potential function. Therefore, we create a novel manually curated data set of plastid proteins and build an ensemble model for prediction of protein subplastid localization. Moreover, we discuss problems associated with the task, e.g. data set sizes and homology reduction. PlastoGram classifies proteins as nuclear- or plastid-encoded and predicts their localization considering: envelope, stroma, thylakoid membrane or thylakoid lumen; for the latter, the import pathway is also predicted. We also provide an additional function to differentiate nuclear-encoded inner and outer membrane proteins. PlastoGram is available as a web server at https://biogenies.info/PlastoGram and as an R package at https://github.com/BioGenies/PlastoGram. The code used for described analyses is available at https://github.com/BioGenies/PlastoGram-analysis.

## Introduction

Plastids play an essential role in sustaining life on Earth. Using CO$_2$ and light in the process of photosynthesis, they produce energy to synthesize carbohydrates, fatty acids and amino acids, as well as oxygen – a byproduct of the reaction. The most well-known type of plastids is the chloroplast, i.e., the photosynthetic organelle of green plants. The relevance of chloroplast capabilities and efficiency makes them promising subjects for bioengineering in search of answers to the biggest challenges of our century^1^. Specifically, chloroplasts have been successfully used as environmental sensors for biomolecules, for the production of biopharmaceuticals, e.g. edible vaccines, in synthetic biology to design new biomaterials, and have the potential to satisfy the demand for increased food production^2^.

**Figure 1:**
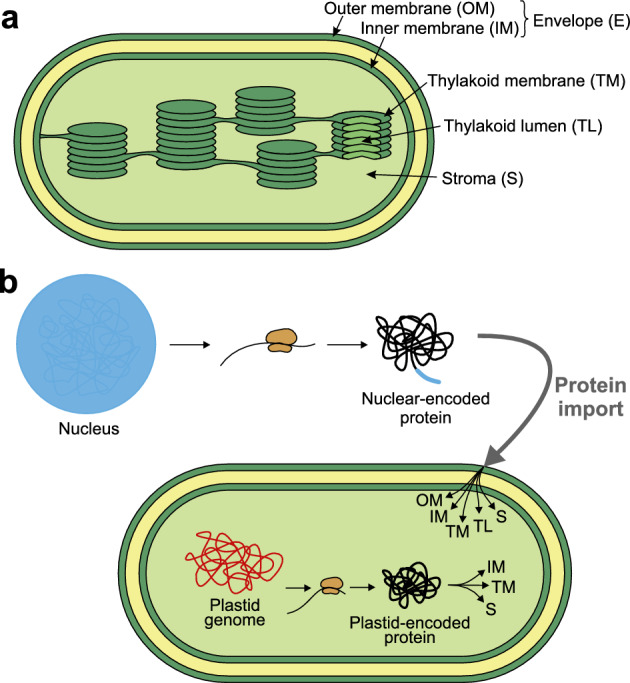
Schematic representation of a plastid structure and plastid protein import. The compartments of plastids considered in this study include the outer (OM) and inner membrane (IM), which together are termed as the envelope (E), stroma (S), thylakoid membrane (TM) and thylakoid lumen (TL) (**a**). Nuclear-encoded proteins are synthesised outside the plastid, in the cytosol, with an N-terminal transit peptide responsible for their targeting to this organelle. They are imported to OM, IM, TM, TL or S. Plastid-encoded proteins are synthesised in the stroma and subsequently transported to IM, TM, or remain in the stroma (**b**).

Chloroplasts are bounded by the envelope (E) composed of the outer and inner membrane (OM and IM) separated by the intermembrane space. The envelope encloses the stroma (S), i.e. an aqueous compartment filling the organelle, and the thylakoids. Thylakoid membranes (TM) contain photosystems responsible for the light-dependent phase of photosynthesis and enclose the thylakoid lumen (TL), another aqueous compartment^3^ (Fig. 1a). Thylakoid membranes also give origin to plastoglobules, which are spherical lipoprotein complexes containing enzymes and structural proteins. They are considered a separate chloroplast compartment but remain physically coupled to thylakoids^4^.

Plastid genomes are highly reduced and encode only about 100 proteins. The majority of proteins required for their proper functioning are encoded in the nuclear genome. It is estimated that 2000-3000 proteins synthesized in the cytosol have to be imported into the chloroplast^5^ (Fig. 1b). Targeting of proteins into their final location occurs thanks to the presence of specific signals within protein sequences. For many chloroplast proteins, such features have been identified, revealing a vast variety of targeting signals (Fig. S1). However, some of the signals may be characteristic of only a single protein e.g., the bipartite presequence of Toc75^6^, or specific groups of proteins e.g., signal-anchored proteins of OM^6^. They also depend on the origin of protein (plastid- or nuclear-encoded), e.g. nuclear-encoded proteins possess transit peptides responsible for their import via the plastid envelope. However, the exact import mechanism has been studied only for a fraction of proteins, so many targeting signals and pathways may still be unknown.

The fact that protein location is tightly associated with its function makes the prediction of their suborganellar localization very desirable. It is reflected by the number of software created for the prediction of subchloroplast location^7–17^, recently reviewed by Liu et al.^18^. The first programs were designed to differentiate only between four compartments and trained on very small data sets (Table 1, S1). Over the years, the number of available plastid sequences have increased, leading to the development of models differentiating between more locations and accounting for the fact that some proteins may be located in more than one subplastid compartment. The first multi-label algorithm was published in 2015 and introduced plastoglobule as another location (Table 1, S1). Noteworthy, only one of the currently available programs, SChloro, can differentiate between six subplastid compartments, i.e. additionally classifies envelope proteins into those located in IM and OM. It is also the only software with architecture influenced by biological properties of proteins, e.g., the presence of a transit peptide or membrane interaction.

**Table 1:** Homology reduction methods and numbers of sequences used by available software. Abbreviations: HR - homology reduction, HP - homology partitioning, TM - thylakoid membrane, TL - thylakoid lumen, IM - inner membrane, OM - outer membrane. *Authors report that the threshold was set to 25% but they use data from MultiP-Schlo created with 40% cutoff.

Software	Year	Homology reduction strategy (cutoff)	Final number of sequences	Envelope	Stroma	TM	TL	Plastoglobule
SubChlo	2009	CD-HIT HR (60%)	262	40	49	129	44	-
ChloroRF	2010	CD-HIT HR (60%)	261	40	49	128	44	-
SubIdent	2011	CD-HIT HR (60%)	262	40	49	129	44	-
BS-KNN	2012	CD-HIT HR (60%)	253	40	46	127	40	-
ChloPred	2013	PISCES HR (25%)	259	77	60	103	19	-
SCLAP	2013	PISCES HR (60%)	341	118	72	111	41	-
WS-LCHI	2014	CD-HIT HR (60%)	262	40	49	129	44	-
MultiP-Schlo	2015	CD-HIT HR (40%)	578	199	105	233	34	30
LNP-Chlo	2016	CD-HIT HR (40%*)	578	199	105	233	34	30
SChloro	2016	HP with PSI-BLAST	367	71 (47 IM, 24 OM)	119	131	37	32
EnTrans-Chlo	2017	CD-HIT HR (40%*)	578	199	105	233	34	30
Bankapur &Patil	2022	CD-HIT HR (40%)	578	199	105	233	34	30

Given a limited number of available sequences, addressing the issue of sequence homology is an essential step in creating a model for subplastid localization. There are two frequently used methods: homology reduction and homology partitioning. The former ensures that no pair of sequences shares identity higher than a given threshold, whereas the latter clusters sequences in a way that their identity percent to sequences in other clusters is lower than a predefined threshold. Homology reduction with 60% identity threshold was applied for data sets used to train all single-label algorithms except ChloPred. The latter employed a 25% cutoff resulting in only 19 proteins in the TL class. The majority of multi-label models were trained on data sets with a maximum of 40% identity. Only one algorithm, SChloro, used a different strategy and utilized homology partitioning to ensure that sequences with local similarity are found in the same set in cross-validation to avoid bias. In all cases, the number of sequences in the most underrepresented class varied from 19 to 40. Consequently, it is worth considering if a model trained on such a small number of sequences can yield reliable predictions, especially taking into account that most algorithms were evaluated using only k-fold cross-validation (CV) or leave-one-out cross-validation (LOOCV) (Table S1). Moreover, in some cases (e.g. LNP-Chlo and EnTrans-Chlo), independent data sets used to evaluate algorithms were not subject to homology reduction due to the small number of sequences, which also questions the credibility of their predictions.

In this project, we wanted to use the increased number of plastid sequences and investigate if it is feasible to create a reliable model with the available data. First, we created a novel manually curated data set of plastid proteins. We focused on biological insights and targeting signals that could increase the model accuracy, i.e. the information if a protein is nuclear- or plastid-encoded and their import pathways. In our opinion, there is still too little data to build a robust multi-label model. Therefore, we focused on predicting one most probable location and additional information about the analysed protein, i.e., its origin and import pathway for TL proteins. The latter may suggest functional or structural properties, e.g., Tat substrates are transported in a folded conformation and might contain cofactors^19,20^.

The result of our research is PlastoGram, an algorithm that allows differentiation of plastid proteins between four compartments (E, S, TL, TM), predicts sequence origin (plastid- or nuclear-encoded) and determines the import pathway for TL proteins. We also discuss problems associated with the prediction of subplastid localization, such as data set sizes, homology reduction and reliability of these predictions. Finally, we suggest directions for future studies, which along with the increase in sequence number and knowledge about mechanisms of protein import could further improve the prediction of subplastid localization.

## Methods

### Data sets

To create data sets of sequences corresponding to compartments of photosynthetic plastids, we searched the UniProt database for proteins annotated as localized in the chloroplast. Importantly, the UniProt keyword ’Chloroplast’ includes not only chloroplasts of green algae and land plants but also plastids of *Rhodophyta*, haptophytes and the SAR supergroup^21^. Therefore, we use the term subplastid localization for sequences of chloroplasts and other types of photosynthetic plastids.

We searched for proteins localized in TL, TM, IM, OM and stroma. For each location, we used separate queries to obtain nuclear- and plastid-encoded proteins. This division is motivated by the fact that protein origin determines its route to the final location, which is reflected in different targeting signals (Fig. S1). We downloaded sequences and their annotations from UniProt release 2021_01^21^ using queries provided in Table S2. We obtained 6198 proteins from chloroplasts of land plants and green algae as well as 746 proteins from other photosynthetic plastids.

We noticed a few mistakes in the annotations concerning the sequence origin, i.e., Toc34 and Toc64 proteins of *Pisum sativum* described as encoded in the plastid genome though they are nuclear-encoded^6^. The list of incorrectly annotated proteins has been reported to UniProt (see Supplementary Methods). We also observed that proteins reported as peripheral membrane proteins in the original articles are sometimes annotated only as membrane proteins in the same way as integral ones. Considering these issues, we manually curated all downloaded sequences and their annotations to ensure the correctness of our data sets. We discarded fragmented sequences, proteins localized in more than one compartment, nuclear-encoded proteins after processing (lacking transit peptides), and those for which we could not find localization evidence in the literature. We also eliminated proteins that localize to plastids and other organelles because due to the scarcity of available data it would introduce too much noise. We grouped peripheral membrane proteins with others localizing in the same aqueous compartments as they are not permanently attached to the membrane. We also decided to exclude a group of 19 plastid-encoded TL proteins because of the insufficient number of sequences and taxonomic representation (only species of rhodophytes and cryptophytes). We did not include plastoglobule as a separate location because there are only 33 plastoglobule proteins identified^22^. Moreover, most of them are also present in other compartments, which is expected considering that plastoglobules originate from thylakoids^22^. Table with accessions, curated locations and references is available in the Supplementary File.

To create final data sets, we separated the thylakoid lumen proteins into two classes according to their import pathway, i.e. Sec or Tat, because each route is associated with the presence of specific, well-characterized targeting signals^20^. Then, we performed additional filtering to prepare data sets for model training. We removed sequences containing amino acids other than standard and reduced the homology of each class-specific data set with CD-HIT^23^ applying the 90% identity threshold and word length 2. The high cutoff value is motivated by the fact that information about protein homology is an important factor in the prediction of subplastid localization. Recent programs utilize this information by performing homology search with, e.g., PSI-BLAST and using obtained PSSMs which hold information about homologous proteins^15–17^. In contrast, we decided to retain more sequences to keep some level of homology information in our data set while still removing the most similar sequences.

**Figure 2:**
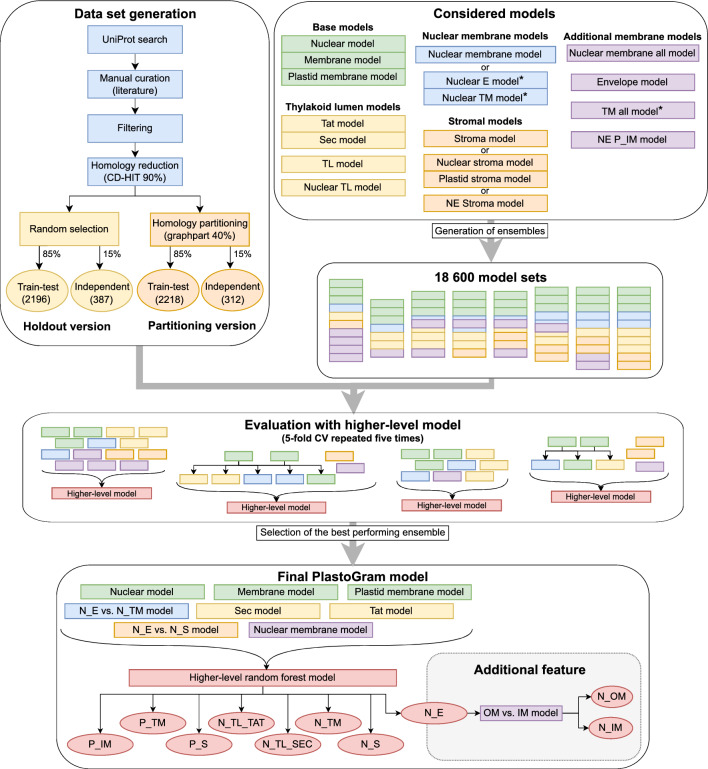
The overall workflow of the best-performing ensemble selection. First, we generated two versions of data sets that differ in the strategy of division between train-test and independent sets. We start with different types of lower-level models designed to deal with specific labeling problems and use them to create model sets. Each set consists of: (i) all base models, (ii) one or both of Sec and Tat models, (iii) none or one of N_TL and TL models, (iv) N_E vs. N_TM model or two class-specific models for prediction of nuclear-encoded membrane proteins, (v) none or one of stroma model, N_S and P_S models, N_E vs. N_S model, (vi) any number of additional models. Next, we built ensembles by combining a set of lower-level models with a higher-level model (random forest or multinomial log-linear model) and specifying information flow between lower-level models (see the Stacked model subsection for details). All ensembles were evaluated in fivefold cross-validation repeated five times on both versions of train-test data sets. The higher-level models were trained on prediction results for folds used to train lower-level models and evaluated on predictions from a test fold. Performance measures were calculated for each fold and replication and then averaged over folds and replications to obtain mean values for each ensemble and data set version. The ensemble that achieved the highest mean kappa value in CV was selected as the final PlastoGram model. Additionally, we provide a separate function to further divide proteins predicted as nuclear-encoded and localized to the envelope into the OM and IM classes. Abbreviations as in Table 2 and S4.

We created two versions of data sets depending on how we divided sequences between the train-test and independent sets. In the first approach (holdout version), we randomly extracted 15% of sequences from each class to create an independent data set for future benchmarking. The rest of the sequences were used in the cross-validation to select the best-performing ensemble (Fig. 2). In the second approach (partitioning version), following the homology reduction, we applied homology partitioning to ensure that independent and train-test data sets do not contain sequences with identity percent above a given threshold. To do that, we have followed the procedure described elsewhere ^24^ with a 40% cutoff, 85%/15% ratio of validation/train-test data sets and without moving between clusters option (Fig. 2). This option disables the relocation of sequences to another partition if there are other sequences within a specified threshold, thus limiting the imbalance between partitions in the case of highly homologous data sets such as plastid proteins. The numbers of sequences at each step are provided in Table 2. We calculated mean pairwise identity percent between train-test and independent data sets for all classes (Table S3) using the Needleman-Wunsch alignment algorithm implemented in needle^25,26^.

**Table 2:** Numbers of sequences in each class at each step of data set preparation. Filtering indicates homology reduction with CD-HIT and removal of sequences with non-standard amino acids, which was performed before the division into two versions of the train-test and independent data sets.

Localization, origin	Dataset	Before filtering	After filtering	Holdout version		Partitioning version	
				Train-test	Independent	Train-test	Independent
Envelope, nuclear-encoded	N_E	118 (59 IM, 59 OM)	115 (59 IM, 56 OM)	98 (50 IM, 48 OM)	17 (9 IM, 8 OM)	96 (50 IM, 46 OM)	10 (6 IM, 4 OM)
Thylakoid membrane, nuclear-encoded	N_TM	276	222	189	33	192	30
Stroma, nuclear-encoded	N_S	357	340	289	51	287	53
Thylakoid lumen, nuclear-encoded(imported via Sec pathway)	N_TL_SEC	49	43	37	6	37	4
Thylakoid lumen, nuclear-encoded (imported via Tat pathway)	N_TL_TAT	84	79	67	12	67	6
Inner membrane, plastid-encoded	P_IM	187	128	109	19	106	11
Thylakoid membrane, plastid-encoded	P_TM	4456	1237	1051	186	1073	156
Stroma, plastid-encoded	P_S	1417	419	356	63	360	42
Total number of sequences	-	6944	2583	2196	387	2218	312

### Prediction of subplastid localization

#### Considered models

First, we created a simple multiclass random forest model to use it as a baseline for comparison with more complex ensembles. Baseline was trained to differentiate between all classes at once. We used the random forest algorithm because of its robustness and internal regularization. Moreover, it has been recently shown to outperform deep learning on tabular data^27^. This model was trained without tuning using n-gram presence/absence matrix as a sequence feature representation. N-grams are short amino acid motifs and may be continuous or with gaps, where gap means any amino acid. We used n-grams of lengths from 1 to 3. For those of length 2, we considered gaps ranging from 1 to 3, whereas for n-grams of length 3, only gaps of length 1 were allowed.

Next, we built separate machine learning models for each labeling problem. The list of considered models with the data subset used for their training is provided in Table S4. We trained both general models, such as for prediction of sequence origin (Nuclear model) or membrane proteins (Membrane model), as well as class-specific models, e.g. Plastid membrane model for discrimination between plastid-encoded TM and IM proteins.

For the prediction of all locations except TL, we used random forest models based on n-grams. Sequence features were represented in a form of n-gram presence/absence matrix. We extracted n-grams of the same length as in the baseline model. Then, we used the quick permutation test (QuiPT)^28^ with a *p*-value cutoff 0.01 for feature selection. Such threshold allowed removal of the insignificant n-grams while keeping sufficiently large feature space for random forest training. The most informative n-grams were then used to build machine learning models using the random forest algorithm from the ranger R package^29^. The numbers of informative n-grams used to train each model are listed in Table S5 and overlap in features used by holdout and partitioning versions of the same model is shown in Fig. S2. Models trained to diffrentiate between N_E and others, N_TM and others, as well as identifying TM were also tested in a setting with SMOTE algorithm to balance the classes. We performed only over-sampling of the minority class in the training set using the SmoteClassif function from the UBL R package^30^.

To build profile HMM models, we aligned sequences from the Sec and Tat data sets with MAFFT^31^ using the most accurate L-INS-i method. Then, we applied the hmmbuild function from HMMER 3.3 software^32^ to generate HMM profiles of Sec and Tat signals.

#### Stacked model

Our prediction algorithm is a collection of diverse models dedicated to solve a specific labelling problem (a combination of subplastid localization and the origin of a protein). It consists of lower-level models, i.e., random forests and profile HMMs, each designed to answer a specific question, as well as a higher-order model. Such an approach is a type of ensemble learning called model stacking^33^. The idea behind ensemble learning is that the aggregation of predictions from multiple models outperforms the individuals.

Given a set of models (either classifiers or regression models) $\mathscr {S}=\{S_1, S_2, \dots , S_n \}$, that can be extended by transformations like PCA or identity ($f(x) = x$), and an aggregation function *G*, an ensemble model is a pair $(\mathscr {S}, G)$ defined such that each prediction can be defined as:

$$\hat{y} = (G, \mathscr {S})~(x) = G(S_1(x), S_2(x), \dots , S_n(x)),$$

where *x* is an example, and $\hat{y} \in \mathbb {R}$ for regression and $\hat{y} \in \mathbb {Z}$ for classification tasks. Model stacking is a special case of an ensemble model, where the aggregation function *G* is a predictive model.

For each data set version, we tested 336 different variants of lower-level model sets. Then, we further diversified them using five main factors: (i) type of the model for the prediction of nuclear-encoded membrane proteins; (ii) presence of stromal- and thylakoid lumen-specific models; (iii) presence of additional models for the most problematic classes; (iv) presence of the HMM-based models; (v) utilization of SMOTE. We inspected all possible configurations of models within mentioned variants. It resulted in the generation of 18,600 sets of models representing combinations of these five factors (see Fig. 2).

Additionally, we have tested two types of ensembles considering the presence of conditionally restricted models. Here, the conditionally restricted models are models where a protein, instead of being classified by all available models, is classified by the subset of the models based on decision rules derived from the expert knowledge. In other words, a protein is analysed by some models only if it fulfills certain conditions, i.e., is predicted by one of the other models as belonging to a specific class. For example, proteins predicted to be encoded in the nuclear genome are being further processed only by the subset of models for nuclear-encoded proteins.

We also considered two types of higher-level models, i.e., random forests and a multinomial log-linear model. We selected these algorithms because they do not need tuning, are relatively interpretable and have been previously used in model stacking^34–36^. We tested all combinations of ensemble and higher-level model types resulting in 74,400 considered ensembles for each data set version. We did not use model weights as it was the goal of the higher-level model to provide a final prediction based on the results from lower-level models. Due to the difficulty of predicting N_OM and N_IM classes as separate in the whole pipeline, we created an additional model for differentiating proteins predicted as nuclear-encoded and localized in the envelope (for details see Supplementary Methods and Table S6).

### Validation and benchmark

To select the best ensemble, we performed a stratified fivefold CV using train-test part of each data set version (for details see Supplementary Methods). The fivefold CV procedure was repeated five times to ensure reliable accuracy estimates. We calculated mean values of kappa, average 1 vs. 1 multiclass AUC (AU1U) and class-specific accuracies (all measures are defined in Supplementary Methods). For the final model we selected the ensemble yielding the highest kappa, as it is a robust measure of the chance-corrected agreement^37^.

The best-performing ensemble (hereafter termed PlastoGram) in each version was then trained on the whole train-test data set and evaluated using sequences from the independent set, which were not used in any earlier steps of the analysis. To assess the performance of our algorithm, we calculated kappa, AU1U and class-specific accuracies.

We compared the performance of PlastoGram with SChloro^17^, one of the latest multi-label programs, which was proven to outperform all algorithms created before its publication ^17^. We were not able to perform benchmark with EnTransChlo^15^ and LNP-Chlo^16^ as their web servers seemed to experience technical problems when provided with multiple sequences, even though they should be able to handle up to 10 sequences at once. The web server of Bankapur &Patil software^38^ also seemed to accept only a single sequence because when provided with a multi-fasta query it returned a single prediction result. Moreover, none of these algorithms is available in a standalone version. We also did not compare our tool with a simple homology search, e.g. BLAST, because this approach has been shown previously to be outperformed by predictive models^10^.

We used the standalone version of SChloro with the current release of the UniProtKB/SwissProt (2022_01) database. We run predictions on both versions of the independent data set and evaluated results in terms of generalized classes: envelope, stroma, thylakoid membrane and thylakoid lumen to make the comparison possible. For example, if a protein was predicted to be OM or IM by SChloro, it was considered an envelope prediction. Similarly, in the case of PlastoGram, the envelope class was assigned to proteins predicted as N_E or P_IM. Moreover, as SChloro is a multi-label model, we considered a protein as properly predicted even if it was assigned to multiple locations, provided that one of them was the correct one.

## Results and discussion

PlastoGram for both data set versions (H–holdout, P–partitioning) had the same architecture. It consisted of eight lower-level models (see Table S4 or Fig. 2 for details) and a random forest higher-level model. It yielded mean kappa 0.869 and AU1U 0.791 for the H version and mean kappa 0.875 and AU1U 0.800 for the P version in repeated cross-validation. Class-specific mean accuracies obtained by both versions of PlastoGram in CV are presented in Fig. S3 with the highest values for P_TM, P_IM, P_S, N_S, and the lowest for N_E. Statistical analysis using Kruskall-Wallis test confirmed consistency of the results obtained by both versions for all classes except N_E and N_TM (*p* = 0.0008 and *p* = 0.0184, respectively; Table S9).

Investigation of the performance measure distribution for all ensembles and the naive baseline model in CV shows that there is indeed a necessity for more complex architecture to correctly predict some classes (Figs. S4, Tables S7–S8). The high imbalance of the data set sizes causes the naive baseline models to obtain a high AU1U (H: 0.818, P: 0.822), which is associated with their ability to predict the most abundant classes; however, they completely fail for less frequent classes (0.03 accuracy for N_E in both versions). Differences in kappa, a more robust metric, show the actual gain from ensembles (baseline H: 0.229, PlastoGram H: 0.869 and baseline P: 0.241, PlastoGram P: 0.875).

On the independent data set, PlastoGram H achieves similar results with kappa 0.893 and accuracies over 0.9 for five classes (Table 3). PlastoGram P performance decreases on the independent data when compared with CV. It yields kappa 0.530 and the accuracies of the least represented classes drop substantially. Such results are expected considering that the overall means of identity percent between the train-test and independent sets are 13.68% and 9.02% for holdout and partitioning versions, respectively. The mean identity percent between the train-test and independent data sets are lower in the partitioning version for all classes (Table S3). Moreover, the partitioning version of the independent data set contained fewer sequences than the holdout, especially for N_TL_SEC, N_TL_TAT and N_E classes with only 4, 6 and 10 cases, respectively. Considering these numbers, it does not necessarily reflect the overall quality of the model predictions.

**Table 3:** Class-specific accuracies obtained in the independent test using 387 (holdout) and 312 (partitioning) sequences and mean values of class-specific accuracies with standard deviations from cross-validation. SD values were not calculated for kappa and AU1U. Abbreviations as in Table 2.

Measure	Holdout version			Partitioning version		
	Independent	Mean	SD	Independent	Mean	SD
kappa	0.893	0.869	-	0.530	0.875	-
AU1U	0.790	0.791	-	0.699	0.800	-
N_E accuracy	0.235	0.484	0.114	0.300	0.348	0.085
N_TM accuracy	0.727	0.653	0.059	0.233	0.710	0.074
N_S accuracy	0.980	0.915	0.039	0.736	0.936	0.035
N_TL_SEC accuracy	1.000	0.644	0.167	0.000	0.700	0.150
N_TL_TAT accuracy	0.833	0.707	0.117	0.333	0.714	0.099
P_IM accuracy	0.947	0.969	0.040	0.818	0.949	0.049
P_TM accuracy	1.000	0.997	0.004	0.981	0.998	0.003
P_S accuracy	0.952	0.940	0.032	0.071	0.944	0.021

These results show that with the available number of sequences it is difficult to create a reliable model that would predict all localizations well. Considering the high sequence similarity between plastid proteins, generally accepted identity thresholds for homology reduction reduce some classes to much less than 50 sequences. The necessity of creating an independent data set reduces the number of sequences that can be used for training even more. It makes the prediction of subplastid localization particularly challenging for deep learning. The ability of neural networks to generalize on small data sets has been demonstrated with tabular but not sequence data^39^. Another difficulty for deep learning methods is the sequence length distribution of plastid proteins with the shortest proteins of length below 50 amino acids (e.g., components of photosystem reaction centers, such as psbK, psaJ, psbN, psbF) and the longest with over 1500 amino acids (e.g., translocon component Tic214 and ferredoxin-dependent glutamate synthase).

The best performance is generally achieved for all plastid-encoded proteins with the majority of accuracies in CV and the independent test over 0.94, in some cases even approaching 1 (Table 3). It is probably not only due to a large number of available plastid sequences but also the distinct differences in the amino acid composition of plastid- and nuclear-encoded proteins^40^. Moreover, plastid-encoded proteins localize to fewer possible compartments and possess less complex targeting signals. All these features indeed result in more accurate prediction of localization for plastid-encoded proteins.

**Figure 3:**
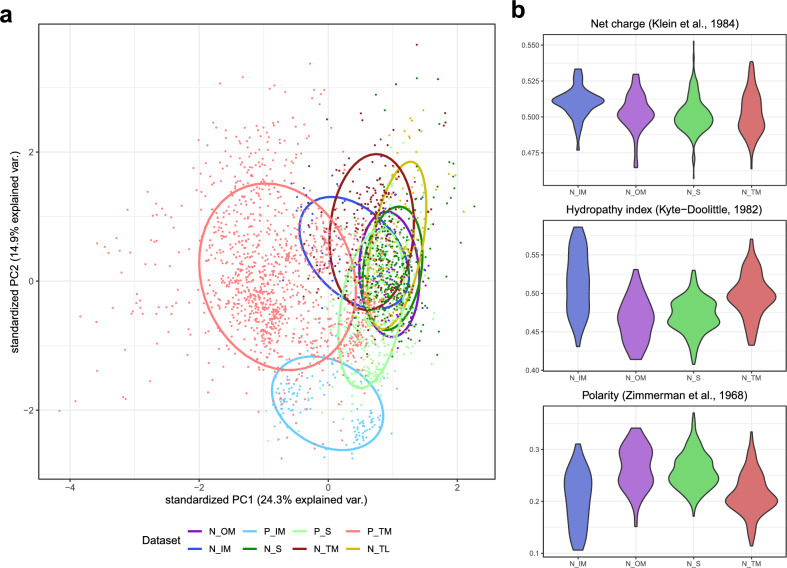
Principal component analysis of the amino acid composition of peptides in each class (**a**). Generally, plastid-encoded proteins form more separated clusters, whereas most of the nuclear-encoded proteins overlap. Noteworthy, the clusters of N_OM and N_S proteins overlap almost completely. Distribution of selected physicochemical properties for nuclear-encoded membrane and stromal proteins in the train-test data set (**b**). OM and stromal proteins follow very similar distribution of all the three properties. IM proteins are characterized by slightly higher net charge and are diverse in terms of mean hydrophobicity and polarity. TM proteins are generally more hydrophobic and less polar than OM and stromal proteins. Abbreviations as in Table 2.

The lowest accuracy is generally obtained for nuclear-encoded envelope proteins. We observed that many of these proteins are incorrectly classified as nuclear-encoded stromal proteins. We performed the principal component analysis of the protein amino acid composition to find the possible cause of these incorrect predictions (Fig. 3a). It shows that generally, plastid-encoded proteins form more separated clusters, whereas most of the nuclear-encoded proteins overlap. Interestingly, N_S and N_OM proteins overlap almost completely. Further investigation of physicochemical properties of the nuclear-encoded membrane and stromal proteins revealed that they are all similar in terms of net charge, hydrophobicity and polarity (Fig. 3b). Noteworthy, there are no statistically significant differences between distributions of these property values for stromal and outer membrane proteins (Table S8).

To check if n-grams capture any known motifs, we analysed the decision rules of n-gram models. Many of the targeting signals of plastid proteins are still unknown, but there are a few described characteristics. One is the DPLG motif found in thylakoid membrane proteins of the light-harvesting chlorophyll-binding protein (LHCP) family^19^. This motif is essential for the interaction of LHCPs with one of the SRP subunits (cpSRP43) and consequently for their import^19^. As LHCPs are nuclear-encoded, we searched for n-grams that are contained within the DPLG motif in the features with the highest Gini importance for Nuclear_membrane model of the PlastoGram P. Out of 4443 informative n-grams used to train this model 13 match the DPLG motif. Moreover, four of them, i.e. DP_G, PLG, D_LG and D_ _G, are among the 50 most important n-grams (Fig. S5). It suggests that n-grams do capture known sequence features and may provide further insights into the process of protein import.

We compared the performance of PlastoGram with SChloro, one of the latest software, on our independent data set. The benchmark results are presented in Fig. S6 and show that PlastoGram outperforms SChloro in all generalized classes except envelope though some of these sequences could have been used in SChloro training. However, it should be noted that SChloro was trained to predict peripheral proteins as located in the membrane, unlike our approach, which might have affected benchmark results to some extent. Interestingly, SChloro seems to exhibit the opposite bias than PlastoGram in the case of envelope proteins. We noticed that many stromal proteins were predicted by SChloro as located in the outer membrane (considered with IM as envelope class in our benchmark). It shows that the issue of high similarity between nuclear-encoded stromal and outer membrane proteins should be considered in future attempts at the prediction of subplastid localization.

Another issue with currently available software is its availability and ability to process large numbers of sequences. Although most of the recently developed algorithms have dedicated web servers, they usually do not allow the analysis of many sequences at once. Moreover, only three of the programs are available in a standalone version. To address this problem, we made PlastoGram accessible as an R package from GitHub, making it easy to implement as a part of the analysis pipelines. It is also available as a web server, which accepts queries up to 50 sequences at once. It is an improvement over EnTransChlo and LNP-Chlo, which allow analyses of up to 10 proteins, and SChloro and Bankapur &Patil, accepting only a single sequence. Another advantage of our tool is its speed, as it does not require the calculation of position specific scoring matrices. Moreover, it introduces means to predict sequence origin and import pathways for thylakoid lumen proteins.

## Conclusion

Our results show that with the currently available sequences we still cannot predict all possible subplastid localizations well, especially IM and OM. We provide a novel manually-curated data set of plastid proteins for future studies while acknowledging that the low number of sequences for some classes not only makes model training challenging but also renders its proper evaluation difficult as it may result in overly optimistic assessment of the performance, especially in the case of holdout version.

Nevertheless, we propose the algorithm developed during this study as an improvement over the existing methods. PlastoGram classifies a protein as nuclear- or plastid-encoded and predicts its most probable localization considering envelope, stroma, thylakoid membrane or thylakoid lumen. For the latter, also the import pathway (Sec or Tat) is predicted. We provided an additional function to differentiate nuclear-encoded inner and outer membrane proteins and made PlastoGram easy to use in different settings, both as a web server and an R package. We implemented both model versions described in the study. Similar to previously published tools, PlastoGram is dedicated to work exclusively on plastid proteins. We did not try to include a step of prediction if a protein localizes to plastids because excellent tools for prediction of subcellular localization already exist, e.g. TargetP^41^ or DeepLoc^42^. Therefore, using PlastoGram for genome annotation requires conjunction with subcellular localization predictors. Considering the already mentioned issues with available software, we believe that PlastoGram offers a lot of new possibilities in the field of plastid protein annotation.

However, there is still room for improvement in the future. New well-annotated sequences (mainly nuclear-encoded), more detailed information about protein targeting signals and novel feature selection techniques should improve the prediction of the most difficult classes, especially the differentiation of outer membrane proteins from stromal ones. Addition of new sequences that localize to multiple compartments or organelles and use of data from large-scale fluorescent tag studies of plastid proteins should also enable development of robust multi-label models. Another issue concerns proteins from other types of photosynthetic plastids than chloroplasts, e.g. rhodoplasts, which represented only 10% of all sequences we collected. Future studies of photosynthetic organisms will provide novel data, which should improve prediction of localization in diverse types of plastids. Moreover, subplastid localization prediction could be extended to multimembrane plastids, including non-photosynthetic plastids of parasitic apicomplexans.

## Supplementary Information

Supplementary Information.

Supplementary Information.

## Data Availability

All data, code and information necessary to reproduce the study are available at https://github.com/BioGenies/PlastoGram-analysis. PlastoGram is available as a web server at https://biogenies.info/PlastoGram and as an R package at https://github.com/BioGenies/PlastoGram.
