# Supplementary material for: Prediction of protein subplastid localization and origin with PlastoGram
Source: Sci Rep. 2023 May 24;13:8365. doi: 10.1038/s41598-023-35296-0 (PMC10209082; doi:10.1038/s41598-023-35296-0)
Supplement: Supplementary file 1 — Supplementary Information. [file 41598_2023_35296_MOESM1_ESM.pdf]

Supplementary Information

Prediction of protein subplastid localization and origin with PlastoGram

Katarzyna Sidorczuk [1], Przemysław Gagat [1], Jakub Kała [2], Henrik Nielsen[3], Filip Pietluch [1], Paweł Mackiewicz [1], Michał Burdukiewicz[4,5]

[1]Faculty of Biotechnology, University of Wrocław, Wrocław, 50-137, Poland,
[2]Faculty of Mathematics and Information Science, Warsaw University of Technology, Warsaw, 00-661, Poland,
[3]Department of Health Technology, Technical University of Denmark, Kgs. Lyngby, 2800, Denmark,
[4]Institute of Biotechnology and Biomedicine, Autonomous University of Barcelona, Cerdanyola del Vallès, 08193, Spain and
[5]Clinical Research Centre, Medical University of Białystok, Białystok, 15-089, Poland.

Methods

Misannotated proteins

1. **Q41009** - Toc34 protein from *Pisum sativum.* Annotated as encoded in the chloroplast genome though it is nuclear-encoded [1].

2. **Q9MUK5** - Toc64 protein from *Pisum sativum.* Annotated as encoded in the chloroplast genome though it is nuclear-encoded [1].

Differentiation between OM and IM

The default version of our model considers nuclear-encoded OM and IM proteins together as envelope class due to the low number of available sequences and the difficulty of predicting these classes as separate in the whole pipeline. However, we see that discrimination between these locations may provide important insights into protein function. Therefore for each data set version, we created an additional model for differentiating proteins predicted as nuclear-encoded and localized in the envelope. The models were trained on N_OM, N_IM and P_IM classes to distinguish OM from IM proteins. Their performance was evaluated in a 5-fold cross-validation repeated five times and the results are provided in Table S6 for both data sets.

Cross-validation

Each localization data set was divided into 5 folds of approximately the same size. Next, one of the folds was kept as a test set, whereas the rest was used to perform feature selection and model training. Obtained models were tested on sequences from the remaining fold. This operation was repeated until each fold has been used as a test set. The whole 5-fold CV procedure was repeated five times to ensure reliable accuracy estimates.

Performance measures

AU1U

AUC of each class against each other, using the uniform class distribution computes the AUC of all possible pairwise combinations.

$$AU1U = \frac{1}{c(c-1)} \sum_{j=1}^{c} \sum_{k=j}^{c} AUC(j,k)$$

where c equals the number of classes, and $f(i,j)$ is the probability of protein i to be of class j.

Kappa

$$Kappa = \frac{P(A) - P(E)}{1 - P(E)}$$

where $P(A)$ is the relative observed agreement among classifiers, and $P(E)$ is the probability that agreement is due to chance [2].

Class-specific accuracy

$$Accuracy(j) = \frac{TP(j)}{TP(j) + FN(j)}$$

where $TP(j)$ is the number of true positives in class j and $FN(j)$ is the number of false negatives in class j. We are aware that generally this measure is termed sensitivity [3]. However, in the subplastid localization prediction field, it is used as a class-specific accuracy [4, 5]. Therefore, we decided to remain consistent with previous studies and also use the term accuracy.

Figures

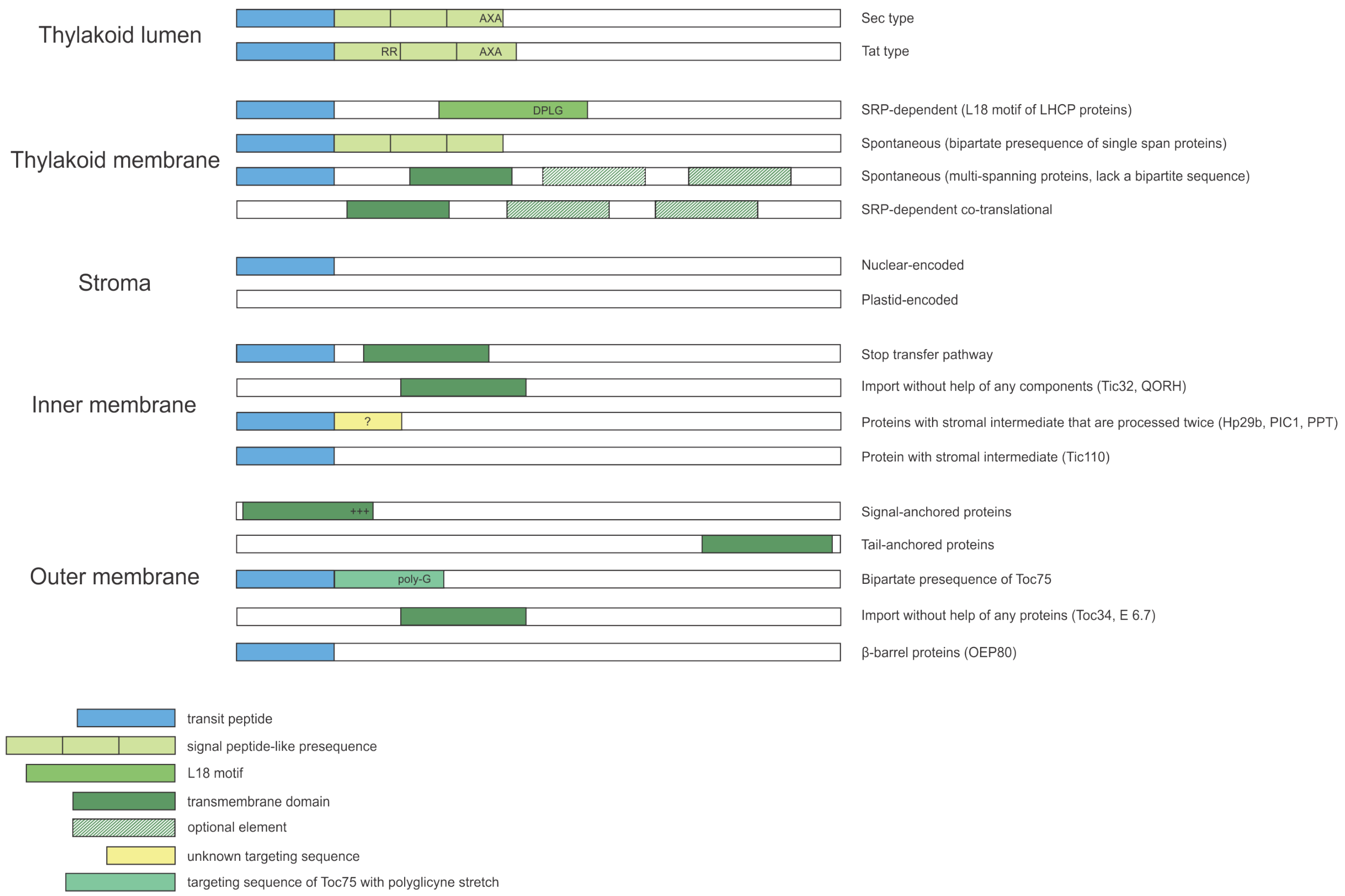

Figure S1: Schematic representation of known signals responsible for protein targeting to various locations. Thylakoid lumen proteins are characterized by the presence of a transit peptide followed by a signal peptide-like sequence. The latter differs depending on the protein import pathway; proteins translocated via the Tat system possess a distinct RR motif [6]. Thylakoid membrane proteins may be targeted via the SRP or spontaneous pathways. The former are mainly Light-Harvesting Chlorophyll a/b Proteins (LHCP) containing the L18 motif or proteins imported co-translationally [7, 8]. Single span proteins that insert spontaneously usually contain a bipartite presequence, which is absent in multi-spanning proteins using this pathway [9, 10]. Stromal proteins do not contain any additional targeting signal except transit peptides in the case of nuclear-encoded ones. Proteins located in the inner membrane may be imported in various ways, e.g. those utilizing stop transfer pathway possess transit peptides and transmembrane domains. Other proteins are imported with stromal intermediate and possess transit peptides, some of them may be processed twice but their targeting sequences are unknown [11]. There is also a group of inner and outer membrane proteins that are imported without the help of any components [12]. The other outer membrane proteins may be signal- or tail-anchored, possess a bipartite presequence, or contain transit peptides [1].

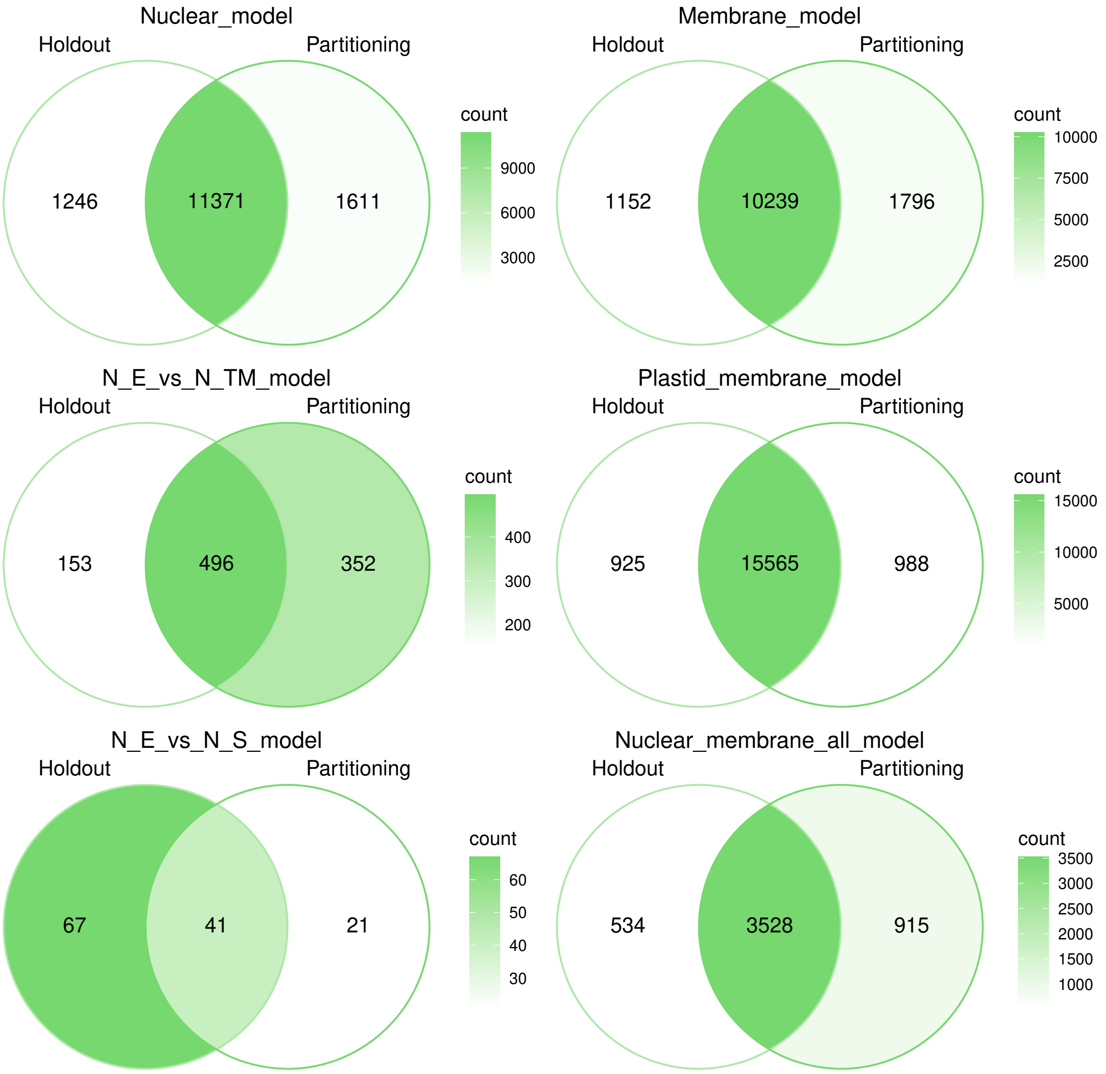

Figure S2: Numbers of features unique and overlapping between holdout and partitioning versions of the same model.

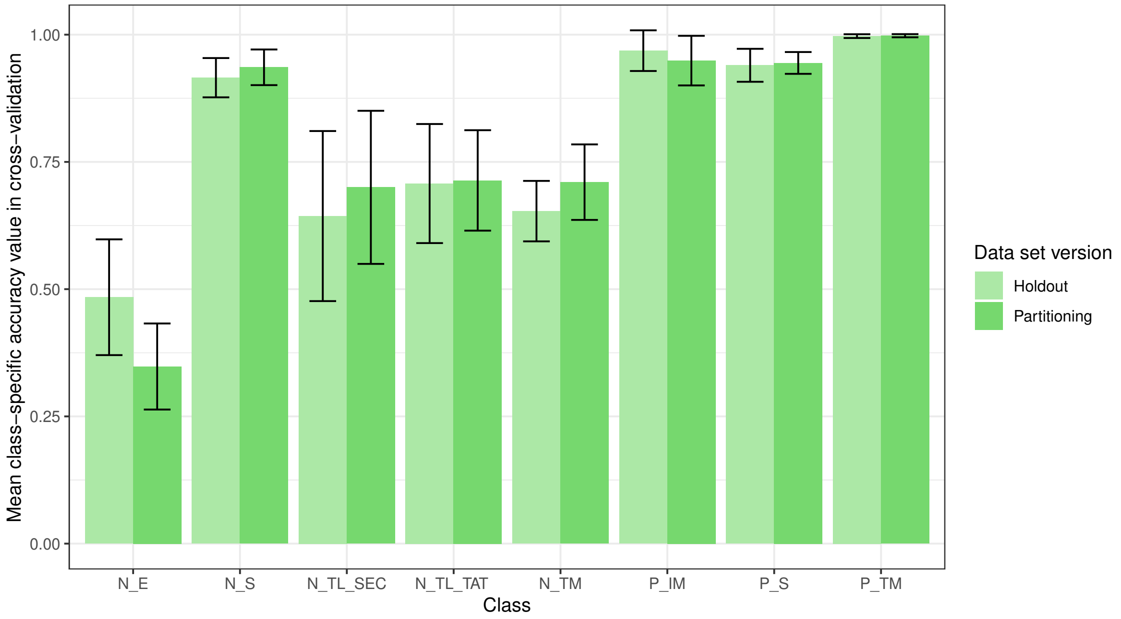

Figure S3: Mean class-specific accuracies obtained in 5-fold cross-validation repeated five times for the best-performing model trained on two versions of data sets.

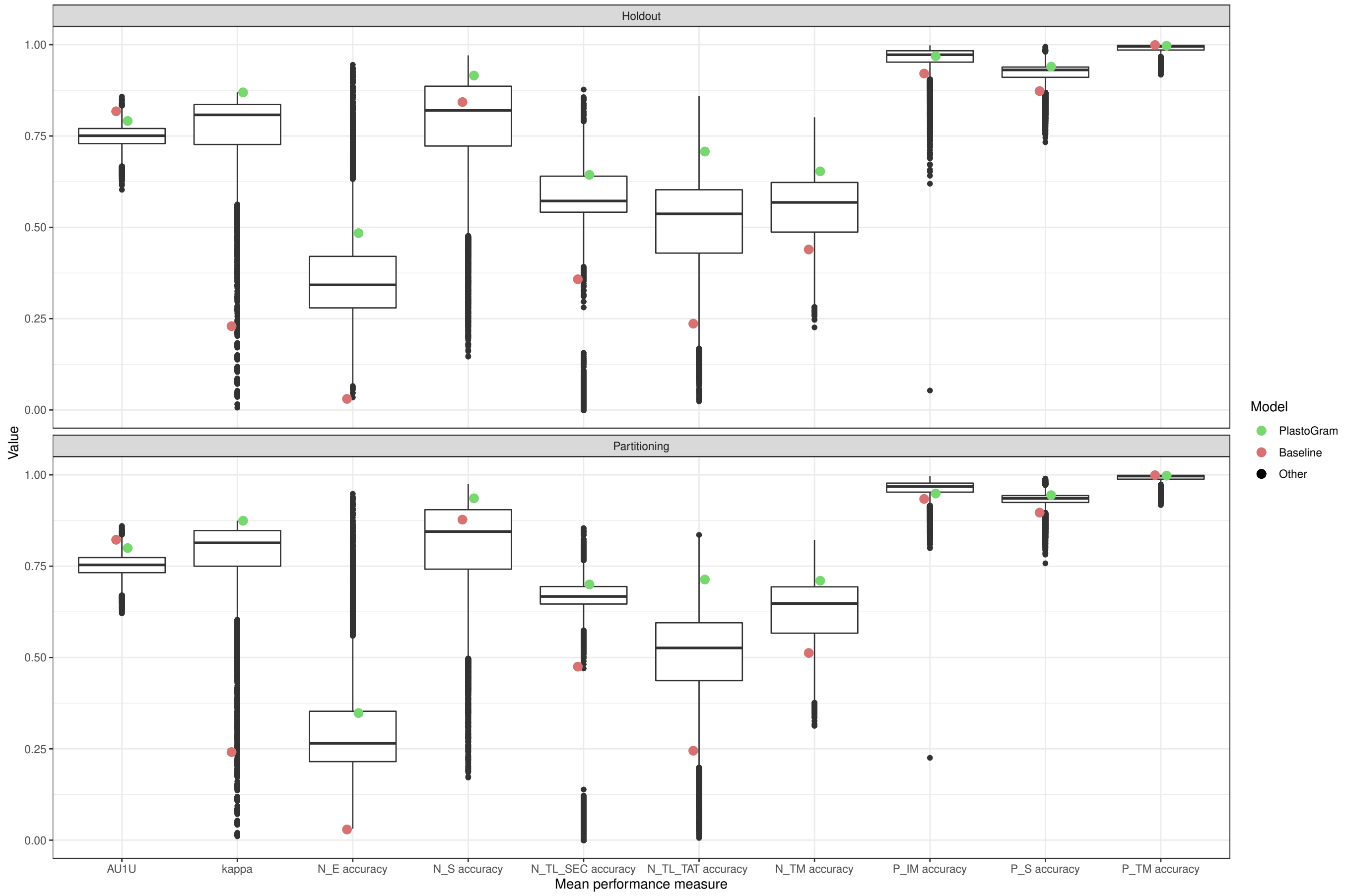

Figure S4: Distribution of performance measures for all investigated ensembles and baseline model (red dots) trained on both versions of data sets. The best-performing ensemble selected as the final PlastoGram model is marked with green dots. Introduction of architectures offers a great improvement in prediction of difficult classes, such as N_E, N_TL_SEC, N_TL_TAT, and the overall performance indicated by kappa.

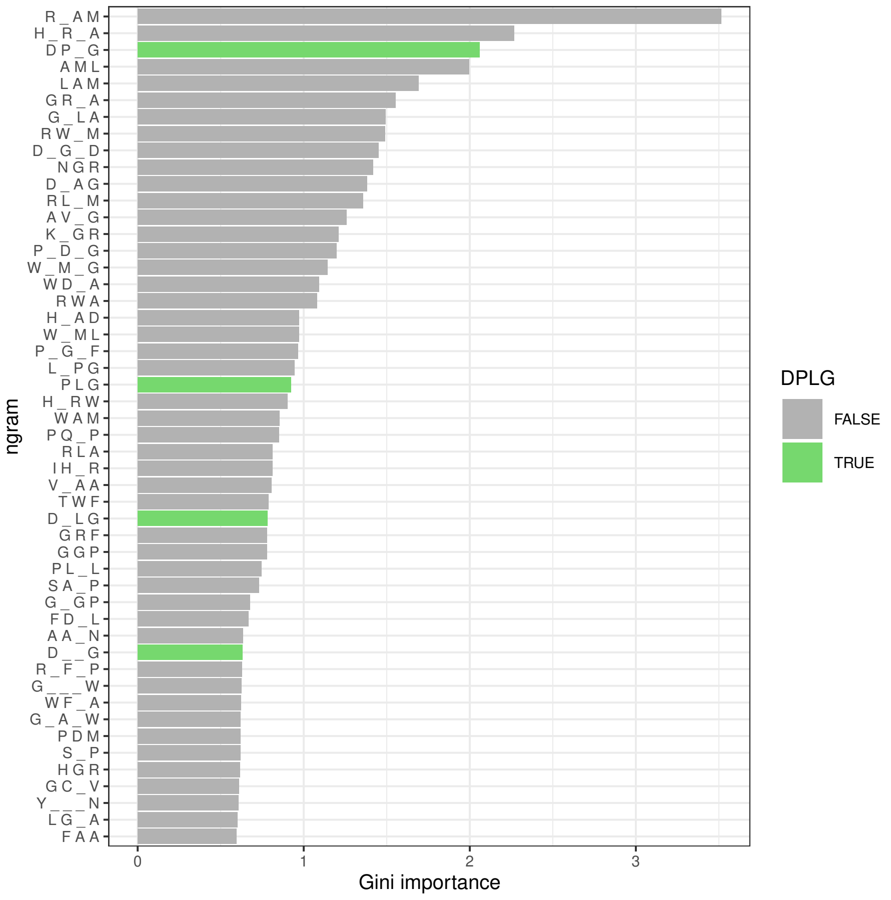

Figure S5: 50 ngrams with the highest Gini importance in Nuclear_membrane model (PlastoGram P) out of all 4443 ngrams used to train this model. Four of the 50 most informative ngrams are contained within the DPLG motif characteristic of thylakoid membrane proteins imported via SRP pathway. Gini importance of these ngrams is marked in green.

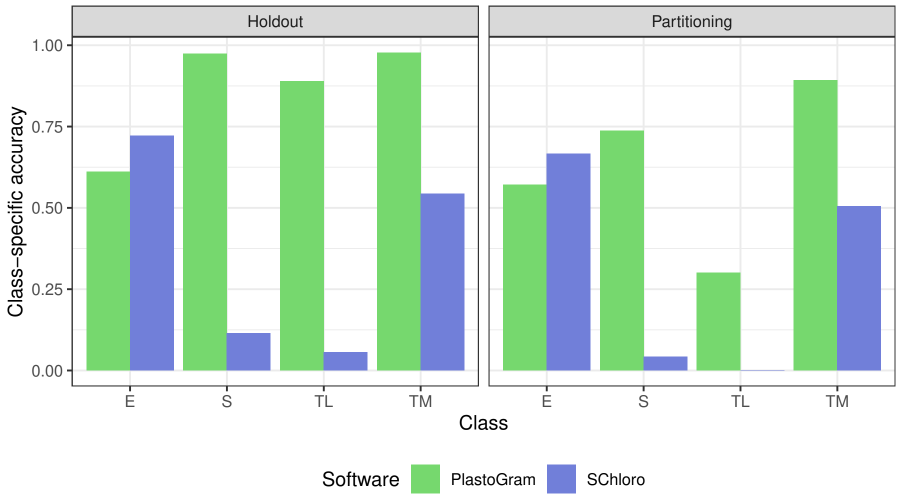

Figure S6: Comparison of PlastoGram and SChloro on both versions of our independent data set considering generalized classes (E - envelope, S - stroma, TL - thylakoid lumen, TM - thylakoid membrane).

Tables

Table S1: Available software for prediction of subchloroplast localization.

Year	Software	Localizations	Multi-label	Stacked	Ensemble	Validation method	Algorithm	Reference
2009	SubChlo	envelope, stroma, TM, TL	no	no	no	LOOCV and 5-fold CV	evidence-theoretic K-nearest neighbor	[13]
2010	ChloroRF	envelope, stroma, TM, TL	no	no	yes	5-fold CV	Random forest	[5]
2011	SubIdent	envelope, stroma, TM, TL	no	no	yes	LOOCV and independent data set	SVM with RBF kernel	[14]
2012	BS-KNN	envelope, stroma, TM, TL	no	no	no	LOOCV, Nested CV and self-consistency test	Bit-score weighted K-nearest neighbor	[15]
2013	ChloPred	envelope, stroma, TM, TL	no	no	no	LOOCV	SVM with RBF kernel	[16]
2013	SCLAP	envelope, stroma, TM, TL	no	no	yes	LOOCV, 5-fold CV and independent data set	AdaBoost	[17]
2014	WS-LCHI	envelope, stroma, TM, TL	no	no	no	LOOCV, independent data set	SVM with RBF kernel	[18]
2015	MultiP-Schlo	envelope, stroma, TM, TL, plastoglobule	yes	yes	yes	LOOCV and resampling*	SVM	[19]
2016	LNP-Chlo	envelope, stroma, TM, TL, plastoglobule	yes	no	yes	LOOCV and independent data set	Multilabel ensemble linear neighborhood propagation	[20]
2016	SChloro	IM, OM, stroma, TM, TL, plastoglobule	yes	yes	yes	LOOCV and 10-fold CV	SVM with RBF kernel	[4]
2017	EnTrans-Chlo	envelope, stroma, TM, TL, plastoglobule	yes	no	no	LOOCV, independent data set, resampling*	Transductive model based on least squares and nearest neighbors	[21]
2022	Bankapur&Patil	envelope, stroma, TM, TL, plastoglobule	yes	no	no	LOOCV and independent data set	Deep neural network	[22]

*20% sequences randomly selected as an independent data set, the rest used for training; procedure repeated 10 times

Table S2: Queries used to perform UniProt searches. Due to the fact that we obtained our data sets using UniProt before the website and search update, we report here queries translated to work with the current UniProt search. The original queries used with the 2021_01 release of UniProt (working on the legacy version, which soon will be shut down) will be provided upon contacting the corresponding author.

Origin	Localisation	Proteins	Query
Plastid	Thylakoid lumen peripheral	13	(cc_scl_term:SL-0058) AND (cc_scl_term:SL-9914) AND (reviewed:true) AND (fragment:false) AND (organelle:plastid)
Nuclear	Thylakoid lumen peripheral	71	(cc_scl_term:SL-0058) AND (cc_scl_term:SL-9914) AND (reviewed:true) AND (fragment:false) NOT (organelle:plastid)
Plastid	Thylakoid lumen	6	(cc_scl_term:SL-0057) AND (reviewed:true) AND (fragment:false) AND (organelle:plastid)
Nuclear	Thylakoid lumen	84	(cc_scl_term:SL-0057) AND (reviewed:true) AND (fragment:false) AND (organelle:plastid)
Plastid	Thylakoid membrane	5218	(cc_scl_term:SL-0058) AND (reviewed:true) AND (fragment:false) AND (organelle:plastid) NOT (cc_scl_term:SL-9914) NOT (cc_scl_term:SL-9917)
Nuclear	Thylakoid membrane	381	(cc_scl_term:SL-0058) AND (reviewed:true) AND (fragment:false) NOT (organelle:plastid) NOT (cc_scl_term:SL-9914) NOT (cc_scl_term:SL-9917)
Plastid	Stroma peripheral (TM)	505	(cc_scl_term:SL-0058) AND (reviewed:true) AND (fragment:false) AND (organelle:plastid) AND (cc_scl_term:SL-9917)
Nuclear	Stroma peripheral (TM)	65	(cc_scl_term:SL-0058) AND (reviewed:true) AND (fragment:false) NOT (organelle:plastid) AND (cc_scl_term:SL-9917)
Plastid	Stroma	273	(cc_scl_term:SL-0055) AND (reviewed:true) AND (fragment:false) AND (organelle:plastid)
Nuclear	Stroma	385	(cc_scl_term:SL-0055) AND (reviewed:true) AND (fragment:false) NOT (organelle:plastid)
Plastid	Stroma peripheral (IM)	1	(cc_scl_term:SL-0051) AND (reviewed:true) AND (fragment:false) AND (organelle:plastid) AND (cc_scl_term:SL-9917)
Nuclear	Stroma peripheral (IM)	6	(cc_scl_term:SL-0051) AND (reviewed:true) AND (fragment:false) NOT (organelle:plastid) AND (cc_scl_term:SL-9917)
Plastid	Inner membrane	190	(cc_scl_term:SL-0051) AND (reviewed:true) AND (fragment:false) AND (organelle:plastid) NOT (cc_scl_term:SL-9917)
Nuclear	Inner membrane	76	(cc_scl_term:SL-0051) AND (reviewed:true) AND (fragment:false) NOT (organelle:plastid) NOT (cc_scl_term:SL-9917)
Nuclear	Outer membrane	84	(cc_scl_term:SL-0054) AND (reviewed:true) AND (fragment:false)

Table S3: Measures of pairwise identity percent distribution between train-test and independent data sets of both versions. Pairwise identity percent values were calculated with needle [23] by comparing all sequences of train-test data with all sequences of the independent set for each location and data set version.

Dataset	Holdout version				Partitioning version			
	Mean	Median	Max	Min	Mean	Median	Max	Min
N_E	8.32	7.60	73.80	0.10	7.79	7.40	27.40	0.00
N_S	10.35	9.80	88.70	0.00	9.09	8.70	39.80	0.00
N_TL_SEC	21.22	13.00	83.90	0.40	13.62	13.20	25.10	0.50
N_TL_TAT	14.45	12.90	83.80	0.30	13.10	13.10	31.50	0.30
N_TM	12.39	10.90	88.80	0.00	9.91	10.00	39.80	0.10
P_IM	23.12	9.20	89.50	0.10	14.15	6.85	40.00	0.10
P_S	11.11	7.00	89.60	0.00	7.52	6.60	32.90	0.00
P_TM	8.44	5.30	90.00	0.00	7.38	5.90	39.50	0.00

Table S4: Lower-level models used in evaluated ensembles with description of their tasks, subsets of data used for training of each model, information if it utilized SMOTE (Synthetic Minority Oversampling Technique) and if it is a part of the final PlastoGram ensemble. Abbreviations as in Table 2.

Model name	Labeling problem	Subset of data used for training	Tested with SMOTE	Present in PlastoGram
Nuclear model	Nuclear-encoded / Plastid-encoded		no	yes
Membrane model	Membrane / Not membrane		no	yes
Plastid membrane model	Plastid-encoded IM / Plastid-encoded TM	Plastid-encoded membrane proteins	no	yes
N_E vs. N_TM model	Nuclear-encoded E / Nuclear-encoded TM	Nuclear-encoded membrane proteins	no	yes
N_E model	Nuclear-encoded E / All others		yes	no
N_TM model	Nuclear-encoded TM / All others		yes	no
Stroma model	Stromal / All others		no	no
P_S model	Plastid-encoded S / All others	Plastid-encoded proteins	no	no
N_S model	Nuclear-encoded S / All others	Nuclear-encoded proteins	no	no
N_E vs. N_S model	Nuclear-encoded E / Nuclear-encoded S	N_E and N_S	no	yes
Tat model	TL imported via Tat pathway / All others	N_TL_TAT	no	yes
Sec model	TL imported via Sec pathway / All others	N_TL_SEC	no	yes
N_TL model	TL / Other nuclear-encoded	Nuclear-encoded proteins	no	no
TL model	TL / All others		no	no
Nuclear membrane model	Nuclear-encoded membrane / All others		no	yes
Envelope model	Envelope / All others		no	no
TM model	TM / All others		yes	no
N_E vs. P_IM model	Nuclear-encoded E / Plastid-encoded IM	N_E and P_IM	no	no

Table S5: Numbers of features, i.e. informative n-grams, used for training of each model.

Model	Holdout	Partitioning
Nuclear_model	12617	12982
Membrane_model	11391	12035
N_E_vs_N_TM_model	649	848
Plastid_membrane_model	16490	16553
N_E_vs_N_S_model	108	62
Nuclear_membrane_all_model	4062	4443

Table S6: Evaluation of the OM vs. IM model trained on the holdout version of the data sets in the 5-fold cross-validation repeated 5 times. Model was trained to distinguish OM from all IM proteins (both N_IM and P_IM), therefore the positive prediction indicates the OM class.

Measure	Holdout		Partitioning	
	Mean	SD	Mean	SD
Kappa	0.651	0.137	0.590	0.164
AUC	0.966	0.025	0.961	0.030
N_OM accuracy	0.613	0.163	0.545	0.197
N_IM accuracy	0.387	0.163	0.455	0.197

Table S7: Parameters of distributions for performance measures obtained in cross-validation of all ensembles on the holdout version of the data sets.

Measure	Minimum	1st quartile	Median	Mean	3rd quartile	Maximum
Mean kappa	0.006	0.727	0.808	0.767	0.836	0.869
Mean AU1U	0.603	0.729	0.751	0.748	0.771	0.858
Mean N_E accuracy	0.034	0.279	0.342	0.361	0.420	0.945
Mean N_TM accuracy	0.226	0.487	0.568	0.555	0.623	0.801
Mean N_S accuracy	0.146	0.723	0.820	0.778	0.886	0.971
Mean N_TL_SEC accuracy	0.000	0.541	0.572	0.568	0.640	0.877
Mean N_TL_TAT accuracy	0.024	0.429	0.537	0.515	0.603	0.860
Mean P_IM accuracy	0.053	0.952	0.972	0.961	0.983	0.998
Mean P_TM accuracy	0.918	0.985	0.995	0.990	0.998	1.000
Mean P_S accuracy	0.733	0.911	0.931	0.919	0.939	0.994

Table S8: Parameters of distributions for performance measures obtained in cross-validation of all ensembles on the partitioning version of the data sets.

Measure	Minimum	1st quartile	Median	Mean	3rd quartile	Maximum
Mean kappa	0.011	0.750	0.814	0.774	0.848	0.875
Mean AU1U	0.621	0.732	0.753	0.752	0.774	0.860
Mean N_E accuracy	0.031	0.215	0.265	0.301	0.353	0.948
Mean N_TM accuracy	0.313	0.567	0.648	0.628	0.693	0.822
Mean N_S accuracy	0.171	0.742	0.845	0.804	0.905	0.975
Mean N_TL_SEC accuracy	0.000	0.646	0.667	0.647	0.694	0.854
Mean N_TL_TAT accuracy	0.006	0.437	0.526	0.509	0.595	0.836
Mean P_IM accuracy	0.225	0.953	0.968	0.960	0.977	0.996
Mean P_TM accuracy	0.917	0.988	0.997	0.991	0.999	1.000
Mean P_S accuracy	0.758	0.924	0.936	0.930	0.943	0.990

Table S9: Statistical analysis of differences between performance of PlastoGram holdout and partitioning versions. Results of the Kruskall-Wallis test with Benjamini-Hochberg correction indicate that there are no statisticaly significant differences in performance measures except N_E and N_TM classes.

Measure	p-value	Adjusted p-value
AU1U	0.1138	0.2276
kappa	0.5671	0.6301
N_E_accuracy	0.0001	0.0008
N_TM_accuracy	0.0037	0.0184
N_S_accuracy	0.1124	0.2276
N_TL_SEC_accuracy	0.1928	0.2754
N_TL_TAT_accuracy	0.9766	0.9766
P_IM_accuracy	0.0413	0.1378
P_TM_accuracy	0.1841	0.2754
P_S_accuracy	0.3685	0.4606

Table S10: Statistical analysis of differences between distributions of physicochemical properties of nuclear-encoded proteins using Mann-Whitney test with Benjamini-Hochberg correction.

Property	Dataset1	Dataset2	p-value	Adjusted p-value	Is significant
Net charge (Klein et al., 1984)	N_OM	N_IM	2.78E-03	4.55E-03	TRUE
Net charge (Klein et al., 1984)	N_OM	N_TM	5.94E-01	6.29E-01	FALSE
Net charge (Klein et al., 1984)	N_OM	N_S	8.31E-02	1.08E-01	FALSE
Net charge (Klein et al., 1984)	N_IM	N_TM	2.42E-04	4.35E-04	TRUE
Net charge (Klein et al., 1984)	N_IM	N_S	1.93E-08	4.34E-08	TRUE
Net charge (Klein et al., 1984)	N_TM	N_S	3.05E-01	3.43E-01	FALSE
Hydropathy index (Kyte-Doolittle, 1982)	N_OM	N_IM	1.33E-08	3.42E-08	TRUE
Hydropathy index (Kyte-Doolittle, 1982)	N_OM	N_TM	5.78E-12	2.60E-11	TRUE
Hydropathy index (Kyte-Doolittle, 1982)	N_OM	N_S	8.42E-02	1.08E-01	FALSE
Hydropathy index (Kyte-Doolittle, 1982)	N_IM	N_TM	8.07E-02	1.08E-01	FALSE
Hydropathy index (Kyte-Doolittle, 1982)	N_IM	N_S	1.17E-11	4.21E-11	TRUE
Hydropathy index (Kyte-Doolittle, 1982)	N_TM	N_S	1.44E-27	1.30E-26	TRUE
Polarity (Zimmerman et al., 1968)	N_OM	N_IM	9.25E-08	1.85E-07	TRUE
Polarity (Zimmerman et al., 1968)	N_OM	N_TM	1.25E-14	7.51E-14	TRUE
Polarity (Zimmerman et al., 1968)	N_OM	N_S	1.17E-01	1.40E-01	FALSE
Polarity (Zimmerman et al., 1968)	N_IM	N_TM	9.60E-01	9.60E-01	FALSE
Polarity (Zimmerman et al., 1968)	N_IM	N_S	1.88E-10	5.64E-10	TRUE
Polarity (Zimmerman et al., 1968)	N_TM	N_S	2.93E-37	5.27E-36	TRUE

References

[1] Hofmann, N. R. & Theg, S. M. Chloroplast outer membrane protein targeting and insertion. *Trends in Plant Science* **10**, 450–457 (2005).

[2] Cohen, J. A coefficient of agreement for nominal scales. *Educational and Psychological Measurement* **20**, 37–46 (1960). https://doi.org/10.1177/001316446002000104.

[3] Powers, D. M. W. Evaluation: From precision, recall and F-measure to ROC, informedness, markedness & correlation. *Journal of Machine Learning Technologies* **2**, 2229–3981 (2011).

[4] Savojardo, C., Martelli, P. L., Fariselli, P. & Casadio, R. SChloro: Directing *Viridiplantae* proteins to six chloroplastic sub-compartments. *Bioinformatics* **33**, 347–353 (2016).

[5] Tung, C.-W., Liaw, C., Ho, S.-J. & Ho, S.-Y. Prediction of Protein Subchloroplast Locations using Random Forests. *World Academy of Science, Engineering and Technology* **65**, 903–907 (2010).

[6] Jarvis, P. & Robinson, C. Mechanisms of Protein Import and Routing in Chloroplasts. *Current Biology* **14**, R1064–R1077 (2004).

[7] Steiner, J. M., Köcher, T., Nagy, C. & Löffelhardt, W. Chloroplast SecE: Evidence for spontaneous insertion into the thylakoid membrane. *Biochemical and Biophysical Research Communications* **293**, 747–752 (2002).

[8] Aldridge, C., Cain, P. & Robinson, C. Protein transport in organelles: Protein transport into and across the thylakoid membrane. *The FEBS Journal* **276**, 1177–1186 (2009).

[9] Robinson, C., Woolhead, C. & Edwards, W. Transport of proteins into and across the thylakoid membrane. *Journal of Experimental Botany* **51**, 369–374 (2000).

[10] Thompson, S. J., Robinson, C. & Mant, A. Dual Signal Peptides Mediate the Signal Recognition Particle/Sec-independent Insertion of a Thylakoid Membrane Polyprotein, PsbY *. *Journal of Biological Chemistry* **274**, 4059–4066 (1999).

[11] Li, M. & Schnell, D. J. Reconstitution of protein targeting to the inner envelope membrane of chloroplasts. *Journal of Cell Biology* **175**, 249–259 (2006).

[12] Firlej-Kwoka, E., Strittmatter, P., Soll, J. & Bölter, B. Import of preproteins into the chloroplast inner envelope membrane. *Plant Molecular Biology* **68**, 505 (2008).

[13] Du, P., Cao, S. & Li, Y. SubChlo: Predicting protein subchloroplast locations with pseudo-amino acid composition and the evidence-theoretic K-nearest neighbor (ET-KNN) algorithm. *Journal of Theoretical Biology* **261**, 330–335 (2009).

[14] Shi, S.-P. *et al.* Identify submitochondria and subchloroplast locations with pseudo amino acid composition: Approach from the strategy of discrete wavelet transform feature extraction. *Biochimica et Biophysica Acta (BBA) - Molecular Cell Research* **1813**, 424–430 (2011).

[15] Hu, J. & Yan, X. BS-KNN: An Effective Algorithm for Predicting Protein Subchloroplast Localization. *Evolutionary Bioinformatics* **8**, 79–87 (2012).

[16] Lin, H. *et al.* Predicting subchloroplast locations of proteins based on the general form of Chou's pseudo amino acid composition: approached from optimal tripeptide composition. *International Journal of Biomathematics* **06**, 1350003 (2013).

[17] Saravanan, V. & Lakshmi, P. SCLAP: An Adaptive Boosting Method for Predicting Subchloroplast Localization of Plant Proteins. *OMICS: A Journal of Integrative Biology* **17**, 106–115 (2013).

[18] Li, X., Wu, X. & Wu, G. Robust feature generation for protein subchloroplast location prediction with a weighted GO transfer model. *Journal of Theoretical Biology* **347**, 84–94 (2014).

[19] Wang, X., Zhang, W., Zhang, Q. & Li, G.-Z. MultiP-SChlo: Multi-label protein subchloroplast localization prediction with Chou's pseudo amino acid composition and a novel multi-label classifier. *Bioinformatics* **31**, 2639–2645 (2015).

[20] Wan, S., Mak, M.-W. & Kung, S.-Y. Ensemble Linear Neighborhood Propagation for Predicting Subchloroplast Localization of Multi-Location Proteins. *Journal of Proteome Research* **15**, 4755–4762 (2016).

[21] Wan, S., Mak, M.-W. & Kung, S.-Y. Transductive Learning for Multi-Label Protein Subchloroplast Localization Prediction. *IEEE/ACM Transactions on Computational Biology and Bioinformatics* **14**, 212–224 (2017).

[22] Bankapur, S. & Patil, N. An Effective Multi-Label Protein Sub-Chloroplast Localization Prediction by Skipped-Grams of Evolutionary Profiles Using Deep Neural Network. *IEEE/ACM Transactions on Computational Biology and Bioinformatics* **19**, 1449–1458 (2022).

[23] Needleman, S. B. & Wunsch, C. D. A general method applicable to the search for similarities in the amino acid sequence of two proteins. *Journal of Molecular Biology* **48**, 443–453 (1970).
